# PneumoBrowse 2: an integrated visual platform for curated genome annotation and multiomics data analysis of *Streptococcus pneumoniae*

**DOI:** 10.1093/nar/gkae923

**Published:** 2024-10-22

**Authors:** Axel B Janssen, Paddy S Gibson, Afonso M Bravo, Vincent de Bakker, Jelle Slager, Jan-Willem Veening

**Affiliations:** Department of Fundamental Microbiology, Faculty of Biology and Medicine, University of Lausanne, Biophore Building, 1015, Lausanne, Switzerland; Department of Fundamental Microbiology, Faculty of Biology and Medicine, University of Lausanne, Biophore Building, 1015, Lausanne, Switzerland; Department of Fundamental Microbiology, Faculty of Biology and Medicine, University of Lausanne, Biophore Building, 1015, Lausanne, Switzerland; Department of Fundamental Microbiology, Faculty of Biology and Medicine, University of Lausanne, Biophore Building, 1015, Lausanne, Switzerland; Department of Genetics, University of Groningen, University Medical Center Groningen, 9713 GZ, Groningen, the Netherlands; Department of Fundamental Microbiology, Faculty of Biology and Medicine, University of Lausanne, Biophore Building, 1015, Lausanne, Switzerland

## Abstract

*Streptococcus pneumoniae* is an opportunistic human pathogen responsible for high morbidity and mortality rates. Extensive genome sequencing revealed its large pangenome, serotype diversity, and provided insight into genome dynamics. However, functional genome analysis has lagged behind, as that requires detailed and time-consuming manual curation of genome annotations and integration of genomic and phenotypic data. To remedy this, PneumoBrowse was presented in 2018, a user-friendly interactive online platform, which provided the detailed annotation of the *S. pneumoniae* D39V genome, alongside transcriptomic data. Since 2018, many new studies on *S. pneumoniae* genome biology and protein functioning have been performed. Here, we present PneumoBrowse 2 (https://veeninglab.com/pneumobrowse), fully rebuilt in JBrowse 2. We updated annotations for transcribed and transcriptional regulatory features in the D39V genome. We added genome-wide data tracks for high-resolution chromosome conformation capture (Hi-C) data, chromatin immunoprecipitation coupled to high-throughput sequencing (ChIP-Seq), ribosome profiling, CRISPRi-seq gene essentiality data and more. Additionally, we included 18 phylogenetically diverse *S. pneumoniae* genomes and their annotations. By providing easy access to diverse high-quality genome annotations and links to other databases (including UniProt and AlphaFold), PneumoBrowse 2 will further accelerate research and development into preventive and treatment strategies, through increased understanding of the pneumococcal genome.

## Graphical Abstract

**Graphical Abstract:**
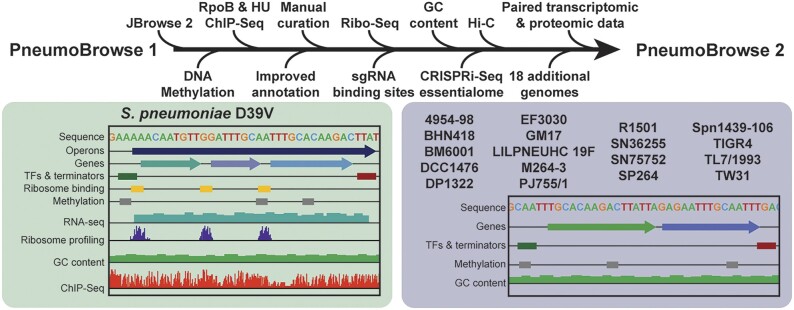

## Introduction

*Streptococcus pneumoniae* is a clinically highly relevant opportunistic human pathogen responsible for over 100 million episodes of lower respiratory tract infections that result in over 500,000 deaths annually. Simultaneously*, S. pneumoniae* is a harmless constituent of the nasopharyngeal microbiome in a large part of the human population (1–3). The balance between lethal opportunistic pathogen or harmless nasopharyngeal commensal is a product of the genomic content and its expression. How the delicate balance between these two lifestyles is controlled is an active area of research. Improved comprehension may come from a more detailed understanding of the regulatory mechanisms surrounding the expression of its genomic contents. Visualizing the genome and its contents, along with data from regulatory and expression studies, can reveal new insights that might otherwise have gone unnoticed.

To understand the gene expression and regulatory networks of *S. pneumoniae*, the oft-used serotype 2 strain D39 (4) and its derivatives such as R6, D39V and D39W have been subjected to extensive DNA and transcriptome profiling studies (5–11). This has resulted in a detailed understanding of the genomic sequence, operon structure, regulatory networks and transcriptome in response to a variety of conditions. These results were bundled in PneumoBrowse (referred to as ‘PneumoBrowse 1″ from here), which was launched in 2018 (5). PneumoBrowse 1 provided an intuitive, user-friendly and visual platform through which the highly detailed manual annotation, including coding features, predicted terminator sequences and repeat sequences, of the circular genome sequence of *S. pneumoniae* D39V could be inspected, but also the in-depth transcriptomic sequencing data. PneumoBrowse 1 thus provided a valuable platform for the pneumococcal research community to gain an in-depth understanding of the genome contents and expression of this important model strain.

Since the release of PneumoBrowse 1, numerous subsequent studies further delved into the genome biology of *S. pneumoniae* using state-of-the-art methods, thereby revealing new complexities in its genome biology. One recently developed method to interrogate pneumococcal gene function on a genome-wide basis has been CRISPR interference (CRISPRi) coupled to next-generation sequencing (CRISPRi-Seq) (12,13). CRISPRi libraries have been used to determine genome-wide essentiality under different conditions ((12,13), and de Bakker and Veening, data to be published). Additional genome-wide works have studied translated features through ribosome profiling (also known as ribosome footprinting, or Ribo-Seq) (14); the correlation between RNA and protein abundances through transcriptomics and proteomics (de Bakker and Veening, data to be published); as well as chromosome conformation and protein binding through high-resolution chromosome conformation capture (Hi-C) and chromatin immunoprecipitation coupled to high-throughput sequencing (ChIP-Seq) experiments (15).

Here, we introduce PneumoBrowse 2, to include the newly available data, but also several major updates. First, we updated the underlying software to JBrowse 2 (16), which vastly improves user experience whilst simultaneously increasing the platform capabilities. Second, we have included the results of the genome-wide Hi-C, ChIP-seq, transcriptomic, proteomic and ribosome profiling studies in D39V. We have also added the binding site locations of the sgRNAs in the CRISPRi library used in recently published work, as well as the gene essentialities determined under different conditions using this library (12,13). In addition, we manually curated and updated gene annotations based on recent molecular studies, and added direct links for each transcribed feature to facilitate the use of other specialized databases, such as UniProt and AlphaFold.

Besides updates to the D39V annotation, we also introduce the genomes of 18 other, phylogenetically diverse, pneumococcal strains in PneumoBrowse 2. Although *S. pneumoniae* strain D39 (derived strains) are among the most investigated strains, other strains are also used to explore pneumococcal biology due to their diverse traits. For example, TIGR4 was the first pneumococcal strain to be entirely sequenced (17), whilst strains BM6001 and DP1322 are used to investigate integrative conjugative element (ICE) biology because they contain ICEs encoding several antibiotic resistance genes (18,19). Strain BHN418 is known for its use in human challenge models (20,21), whilst strains EF3030 and LILPNEUHC 19F are used to investigate the clinically relevant serotype 19F group of *S. pneumoniae* (22). We include the long-read assembled genomes of these and other, phylogenetically diverse, *S. pneumoniae* strains in PneumoBrowse 2. These genomes have been annotated for genomic content, transcription factor bindings sites, Rho-independent terminator sequences and chromosomal DNA methylation patterns.

With these improvements, PneumoBrowse 2 will serve as a portal to the latest insights on the contents and workings of a wide variety of pneumococcal genomes. By making these data accessible for a wide audience, we anticipate that the pneumococcal research field can make additional progress into the biology of these diverse *S. pneumoniae* strains. PneumoBrowse 2 is freely available on https://veeninglab.com/pneumobrowse.

## Materials and methods

### Strains used in this work

The strains included in PneumoBrowse 2 are listed in Table 1.

**Table 1.** *S. pneumoniae* strains presented in PneumoBrowse 2

Pneumobrowse ID	Alternative IDs	MLST	Serotype	SRA accession	Genome accession	Origin	Reference strain	Remark	Reference
4954–98	Tennessee14-18, ATCC BAA-340	67	14	SRR28781549	JBDIMH000000000	*NA*	Yes; PMEN18 reference strain		(77)
BHN418	*NA*	138	6B	SRR28790986	CP155538	*NA*	No		(78)
BM6001	*NA*	Unknown	19F	SRR28781558	JBDIMJ000000000	*NA*	No	Carries Tn5253 composite integrative conjugative element.	(18)
D39V	NCTC 14078	595	2	*See Slager et al., Nucleic Acids Research, 2018*	*See Slager et al., Nucleic Acids Research, 2018*	*NA*	No	NCTC 7466 derivative	(5)
DCC1476	Sweden15A-25, ATCC BAA-661	156	15A	SRR28781547	CP155534	*NA*	Yes; PMEN25 reference strain		(79)
DP1322	*NA*	Unknown	*unencapsulated*	SRR28781557	CP155537	*NA*	No	R6 (unencapsulated D39 derivative) derivative carrying Tn5253	(18)
EF3030	*NA*	43	19F	SRR28781559	JBDIMI000000000	*NA*	No		(80)
GM17	Spain 6b-2, ATCC 700670	90	6B	SRR28781552	CP157351	*NA*	Yes; PMEN2 reference strain		(81)
LILPNEUHC 19F	*NA*	179	19F	SRR28781556	JBDIMK000000000	Blood isolate from patient with invasive pneumococcal disease.	No		This study
M264-3	Netherlands3-31, ATCC BAA-1657	180	3	SRR28781545	CP155536	*NA*	Yes; PMEN31 reference strain		(82)
PJ755/1	Sweden1-28, ATCC BAA-1655	306	1	SRR28781546	CP155535	*NA*	Yes; PMEN28 reference strain		(83)
R1501	*NA*	5954	*unencapsulated*	SRR29015166	CP155800	*NA*	No	R6 derivative. No modified bases resulted from analysis of the PacBio sequencing data due to insufficient quality of SMRT Link-hits.	(84)
SN36255	*NA*	320	19A	SRR28781561	CP155531	Blood isolate from patient with invasive pneumococcal pneumonia.	No		This study
SN75752	*NA*	6521	11A	SRR28781553	*see Gibson et al., Plos Pathogens, 2022*	*NA*	No	Referred to as "11A" in reference. No modified bases resulted from analysis of the PacBio sequencing data due to insufficient quality of SMRT Link-hits.	(76)
SP264	Spain 23F-1, ATCC 700669	81	23F	SRR28781560	CP155532	*NA*	Yes; PMEN1 reference strain		(81)
Spn1439-106	Colombia 5–19, ATCC BAA-341	289	5	SRR28781548	CP155533	*NA*	Yes; PMEN19 reference strain		(85)
TIGR4	*NA*	205	4	SRR28781554	CP155539	*NA*	No		(76)
TL7/1993	Spain 9V-3, ATCC 700671	156	9V	SRR28781551	CP156625	*NA*	Yes; PMEN3 reference strain		(81)
TW31	Taiwan19F-14, ATCC 700905	236	19F	SRR28781550	JBDIMG000000000	*NA*	Yes; PMEN14 reference strain		(81)

The origin of the strain is noted if no previous reference is available. Strains that serve as a reference clone for one of the 43 clones defined by the Pneumococcal Molecular Epidemiology Network (https://www.pneumogen.net/gps/#/resources#pmen clones) are indicated as such.

No MLST type could be defined for strains BM6001 and DP1322 through mlst. Strains DP1322 and R1501 are derivatives of S. pneumoniae strain R6 and do not encode a functional capsular polysaccharide biosynthesis gene cluster in their genomes and are thus unencapsulated.

The analysis of PacBio sequencing reads for methylated bases through SMRT Link did not yield results for strains R1501 (only bases with unspecified tag ‘modified_base’) and SN75752 (none of the SMRT Link called hits passed quality filtering parameters). NA; not applicable.

The strain names used in PneumoBrowse 2, alternative identifiers(s), multilocus sequence type, serotype and accession codes for the genomic and sequencing data deposited at the Sequence Read Archive (SRA).

### PneumoBrowse 2 web interface

PneumoBrowse 2 is available online (https://veeninglab.com/pneumobrowse) through JBrowse 2 (16). The NucContent plugin for JBrowse2 is used to provide a track to calculate and visualize GC content (available from https://github.com/jjrozewicki/jbrowse2-plugin-nuccontent).

### Isolation of genomic DNA

Genomic DNA was isolated through previously published methods, with minor adaptations (23,24). Bacterial cultures were grown to an optical density at 595 nm of 0.2 in C + Y medium (24) without shaking, spun down and resuspended in Nuclei Lysis solution containing 0.05% SDS, 0.025% deoxycholate and 200 μg/mL RNase A. The suspension was incubated at 37°C (20 min), 80°C (5 min), 37°C (10 min) and cooled to room temperature. Lysates were treated with Protein Precipitation Solution (Promega), vortexed vigorously, incubated on ice for 10 min, then pelleted by centrifugation. DNA was precipitated from supernatants with isopropanol and collected by centrifugation. Pellets were washed once in 70% ethanol, air-dried and resuspended in water.

If isopropanol failed to produce sufficient DNA, FastPure DNA Isolation Mini Kit columns (Vazyme) were used. After protein precipitation and centrifugation, supernatant was transferred to the column and processed according to protocol. DNA was eluted off the column in water.

### Whole genome sequencing and assembly

DNA concentrations were quantified with a Qubit 4.0 fluorometer, using the high-sensitivity double-stranded DNA kit. Quality control for chromosome integrity was performed by running the gDNA on a 1% agarose gel, and through an Agilent Fragment Analyzer.

Long-read sequencing for 4954-98, BM6001, DCC1476, DP1322, EF3030, GM17, LILPNEUHC 19F, M264-3, PJ755/1, R1501, SN36255, SP264, Spn1439-106, TL7/1993 and TW31 was performed using a PacBio Sequel II machine. PacBio reads were demultiplexed, filtered for quality and assembled through SMRT Link (25).

Long-read sequencing for BHN418 was performed using an Oxford Nanopore MinION Mk1B, the Rapid Sequencing Kit V14 and an R10.4.1 flow cell. Nanopore POD5 files were base-called using Dorado (v0.6.0; available from https://github.com/nanoporetech/dorado) using the ‘super accurate basecalling model’. Resulting FASTQ files were used for assembly using Unicycler (v0.5.0) (26).

### Genome annotation and characterization

Assembled genomes were annotated through Prokka (v1.14.6) (27). The Prokka script was adapted by removing the ‘-c’ flag from Prodigal (28), to enable annotation of open reading frames without a start or stop codon at the ends of a contig. Aragorn (v1.2.38) was used for the annotation of transfer RNAs (29); Infernal (v1.1.4) and Rfam were used to annotate non-coding RNAs (30); Minced (v0.4.2) was used to identify CRISPR arrays (31); and HMMER3 (v3.3.2) was used for protein similarity searching (32). The previously produced high-detail annotation of D39V was used as reference (5). Rho-independent terminators were predicted through TransTermHP (v2.09) (33), and filtered for a minimal score of 60. ComE-, ComX- and ParB-binding sites were determined through FIMO (MEME suite v5.5.5), using the previously determined consensus sequences in *S. pneumoniae* D39V (5,7,34,35), and filtered based on their q-values (q ≤ 0.001 for ComE and ComX; q ≤ 0.01 for ParB).

Modified bases were called from the PacBio reads using SMRT Link and filtered for a QV score ≥ 100. (Hydroxy)methyl modifications were determined from Nanopore POD5 reads using Dorado (v0.6.0) using the ‘super accurate model’, extracted using modkit (v0.2.7; available from https://github.com/nanoporetech/modkit), and filtered based on the number (≥20), and fraction (≥95%) of reads supporting the modification.

Additional functional annotation for the D39V genome was performed using the online eggNOG-mapper (v2) tool (36–40).

MLST typing for all genomes was performed using mlst (v2.23.0; available from https://github.com/tseemann/mlst).

### Genome comparison and core genome phylogenetic analysis

Closely related genomes were aligned using the command-line version of progressiveMauve (version of February 13, 2015) (41).

A core genome alignment for all genome sequences included in PneumoBrowse 2 and genomes representing the phylogeny of *S. pneumoniae* was built using snippy (v4.6.0, available from https://github.com/tseemann/snippy), using D39V as reference (42). Duplicate genomes were filtered out of the genome set from Antic *et al.* Alignments were corrected for recombination using Gubbins (v3.3.1) (43). The phylogeny was constructed using FastTree (v2.1.10) (44) and mid-point rooted and visualized using FigTree (v1.4.5) (available from http://tree.bio.ed.ac.uk/software/figtree/).

Alignment data for inspection within PneumoBrowse 2 was generated by aligning genome data using Mashmap3 (v3.1.3), using the –dense option and setting the identity threshold to 95% (45).

## Results

### Upgrading PneumoBrowse to JBrowse 2

PneumoBrowse 1 was set up using the JBrowse 1 software (46). However, the halted development of JBrowse 1 makes PneumoBrowse 1 increasingly harder to maintain and to update with new data due to outdated libraries and plugins. To remedy this, we updated the underlying software running PneumoBrowse to the newly developed JBrowse 2, which offers significant upgrades compared to JBrowse 1 (16). The upgrade ensures the longevity of PneumoBrowse 2 by accommodating a broader range of data types and offering simplified methods for adding them, making future maintenance more efficient.

The update to JBrowse 2 also provides a significant increase in quality of experience for users. For example, data tracks are now loaded faster, and users may share, restore or export their session, which will preserve the user's current view for future reference. Thus, PneumoBrowse has had a significant increase in performance, user experience and flexibility for future updates with the update to JBrowse 2.

### Further detailing the annotation of the *S. pneumoniae* D39V genome

Since the introduction of PneumoBrowse 1, new studies have further explored the genome biology of *S. pneumoniae*. To reflect the state of the art in pneumococcal genome biology, we have further curated the existing detailed manual annotation of the *S. pneumoniae* D39V. In total, 623 changes to features in the annotation of D39V were made.

Out of these 623 changes, 375 changes were made to the annotations of transcribed features (Supplemental Table S1). The genomic annotation now reflects the latest insights in the genome of *S. pneumoniae* through 230 changes to previously existing annotations of transcribed features. For example, the annotation for SPV_0476 has been updated to code for the pneumococcal spatiotemporal cell cycle regulator CcrZ, and SPV_0878 has been revised to encode the regulator of chromosome segregation RocS, respectively, since the products of these genes have now been shown to play crucial roles in the pneumococcal cell cycle (23,47). Another study has annotated a group of aquaporins at loci SPV_1320 (Pn-*aqpC*), SPV_1569 (Pn-*aqpA*) and SPV_2011 (Pn-*aqpB*) (48). In addition, the functions of SPV_1416 (*murT)* and SPV_1417 (*gatD*) in peptidoglycan biosynthesis have been determined (13).

**Figure 1.**
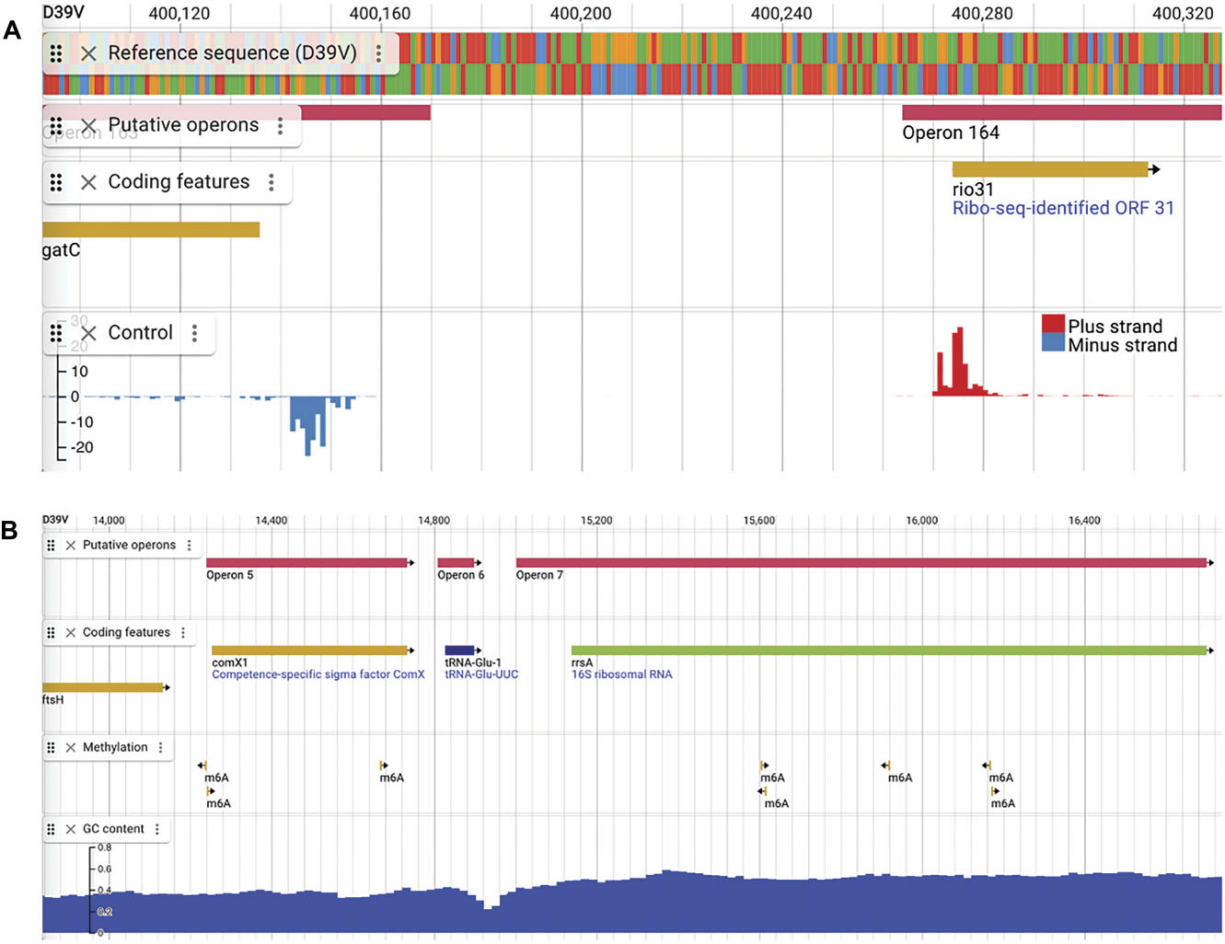
Genome-wide data tracks for D39V in PneumoBrowse 2. (**A**) A genome-wide data track for the normalized intensities of ribosome profiling under control conditions has been added for D39V (14). The data track includes the score for the positive strand (upwards, red), and the negative strand (downwards, blue). (**B**) Genome-wide data tracks for methylated bases in the chromosome of D39V and GC content. The annotations of the methylated sites indicate the type and strand-specificity of the methylated bases. A track for the calculation of GC content (using customizable settings) is provided through the NucContent plugin.

Besides modifying previously existing annotations, we have also added 122 new locus tags to reflect the annotation of previously unknown transcribed features in the D39V genome. Of these 122 locus tags, 114 (SPV_2459 - SPV_2572) are small open reading frames (sORFs), that are the result of the identification of their previously unannotated translational start sites through ribosome profiling (14). These sORFs are now annotated as Ribo-Seq-identified ORF genes (*rio* genes) (Figure 1A, Supplemental Table S2). Four *rio* genes were further annotated: *rio9* and *rio12* were annotated as *rtgS1* and *rtgS2* (49), *rio82* as *shp1518* (50), and *rio3* was shown to be important for nasopharyngeal colonization, although the biological mechanism remains unclear (14). The remaining 110 *rio* genes currently remain without a known biological function. Four additional transcribed features in the *rtg* locus containing peptidase ABC-transporters (SPV_2573 - SPV_2576) (49), and four non-coding RNAs with unknown functions propagated from the TIGR4 annotation in RegPrecise (SPV_2577 - SPV_2580) (51) have also been newly annotated. In addition, the exact coordinates of 17 transcribed features were updated, 15 of which were updated through inspection of the ribosome profiling data and cross-referencing with other databases (14,52). Finally, *srf-01* was changed in coding feature type (from ncRNA to pseudogene) (7), and ncRNA *srf-06* (SPV_2120) was replaced by *shp144* (SPV_2573) (53,54).

Besides 374 changes to transcribed features, 249 changes are made to annotations of features involved in transcriptional regulation, such as transcription start sites, −10 signals, −35 signals, and transcription factor-binding sites (Supplemental Table S2). A total of 60 annotations were added for transcription factor-binding sites, including for those transcription factors crucially involved in competence and transformation, such as ComE and ComX (6,55). Moreover, 31 previously existing transcription factor binding site annotations were modified for their exact location and binding moiety (7,55). Further refinement of transcriptional regulation annotations came from the addition of 104 −10 signals, −35 signals and transcriptional start sites to the D39V annotation through either manual curation of RNA-seq data, or from transcriptional landscape studies of *S. pneumoniae* (7,55). In contrast, 54 annotations previously made through algorithmic analysis have been removed after manual visual inspection of RNA sequencing data (e.g. no appreciable signal in 5′-enriched RNA sequencing coverage or located within repeat regions).

The annotation changes detailed here, have also been incorporated in the GenBank annotation (accession number CP027540.1).

### Addition of genome-wide data tracks for D39V

Individual annotations in the D39V genome give detailed information about the encoded contents of the genomic sequence. However, this does not give insight into the overall regulation and expression of the genome. Besides the binding of transcription factors, gene expression is also influenced through the methylation of chromosomal DNA (56). In PneumoBrowse 2, we have now added a data track to show the position of the methylated bases in the D39V genome, determined previously from PacBio sequencing reads (Figure 1B) (5,56). This resulted in the annotation of 3655 N^6^-methyladenosine bases and three N^4^-methylcytosine bases. For each modified base, the QV score of the call is also available.

**Figure 2.**
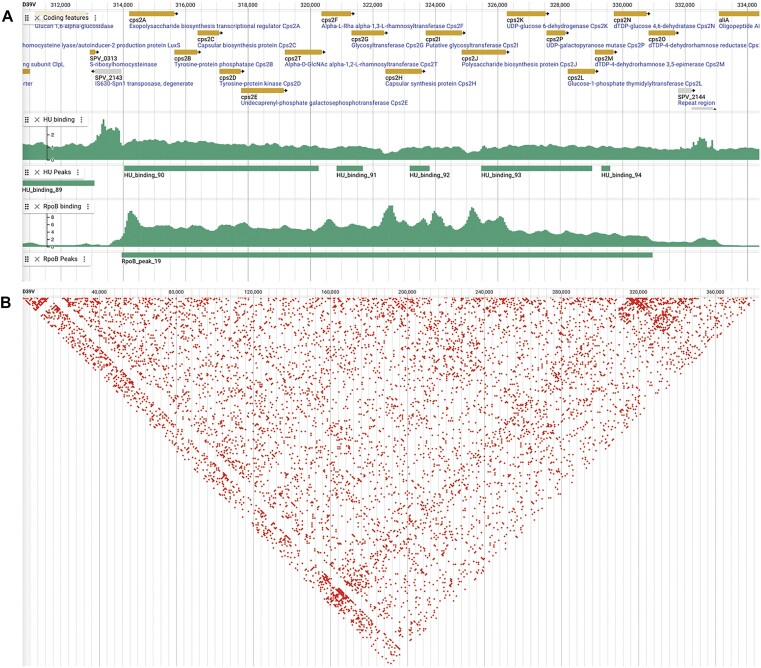
Tracks with ChIP-Seq data for chromosomal protein binding and Hi-C conformation capture data in PneumoBrowse 2. (**A**) Genome-wide data tracks representing ChIP-Seq results for HU and RpoB binding to the chromosome. The plotted values indicate the ratio of the signal between the immunoprecipitated and the control experiments at a resolution of 50 bp. MACS2-called peaks in the RpoB- and HU-binding data indicate regions of enriched binding (15). (**B**) Hi-C chromosome conformation capture data of the first 500,000 nucleotides, with a resolution of 1000 bp, showing the interactions this part of the chromosome on a diagonal line, with interactions marked in red (15).

The binding of RNA polymerase, which consists of five subunits, including the β subunit RpoB, can be influenced by binding of different transcriptional regulators to the chromosome. The binding of RpoB thus indicates sites of active transcription. In addition to RpoB binding, HU (the pneumococcal histone-like protein, also known as HlpA) can influence transcription and replication through its role in chromosomal organization by facilitating condensation. The sites to which RpoB and HU bind to the chromosome of exponentially growing *S. pneumoniae* D39V at 37°C in C + Y medium, have now been determined using ChIP-Seq studies (15). The data from these ChiP-Seq studies are presented in PneumoBrowse 2 as the fold enrichment of immunoprecipitated samples compared to controls (Figure 2A).

Although elucidation of the binding locations of HU through ChIP-Seq studies may indicate the local condensation state of the chromosome, Hi-C experiments are needed to determine which parts of the genome are in close proximity to each other, to define the overall chromosome architecture (57). The novel support of JBrowse 2 data from these types of experiments now allows us to present data on the chromosomal architecture of *S. pneumoniae* D39V (Figure 2B) (15). PneumoBrowse 2 presents the Hi-C data of D39V through a contact heat map in which regions that are observed to be in contact are shaded on the diagonal.

Finally, we have also added a separate track to display the GC content of the genome (Figure 1B). Visual inspection of the GC content can highlight recently horizontally acquired regions such as ICEs or prophages (58). The calculation of the GC content can be adapted through the settings for the NucContent plugin by setting the window size (in bp), window overlap (in percentage) and the calculation method (average, or skew).

### Gene essentiality and expression data under infection(-mimicking) conditions

**Figure 3.**
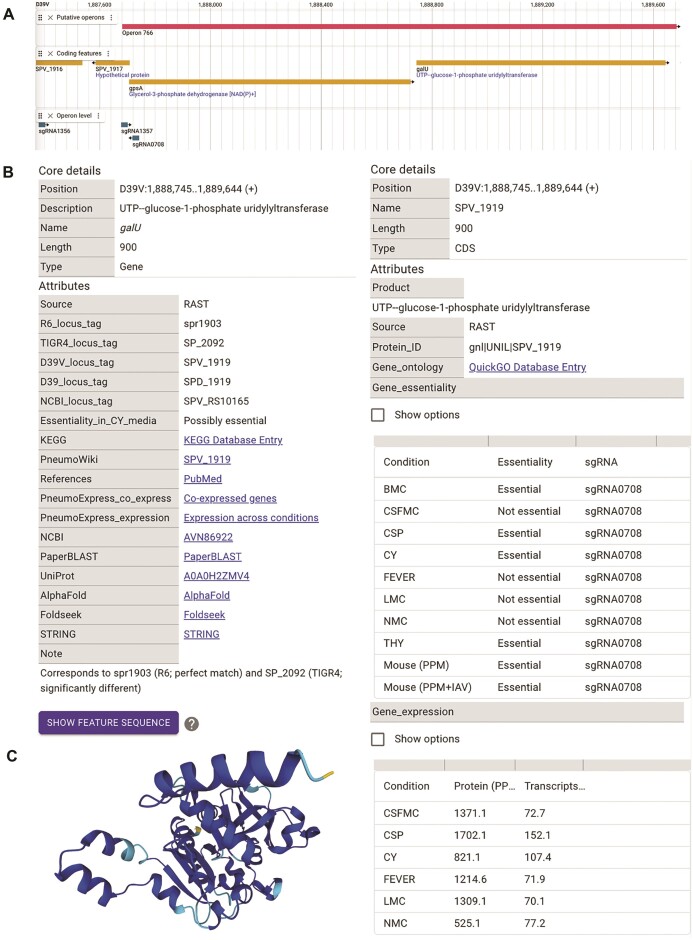
Position of sgRNAs binding sites and detailed information of annotated features for the D39V genome in PneumoBrowse 2. (**A**) A genome-wide data track displaying the position of the sgRNAs included in the CRISPRi-Seq library for D39V (12,13). The positions of the 20-nucleotide long sgRNA spacers are on the non-template strand of the targeted coding feature. (**B**) Overview of the information available for each coding feature in the D39V annotation: the information for *galU* (SPV_1919) is shown. Information for each coding sequence includes links to external resources (AlphaFold, PneumoWiki, UniProt, amongst others), gene essentiality data, and expression data (12) (de Bakker and Veening, data to be published). (**C**) The predicted AlphaFold structure of GalU that is available through the AlphaFold and UniProt links in PneumoBrowse, is shown.

Important outstanding questions in pneumococcal biology include not only how the genome is expressed during infection conditions but also which genes are essential under such conditions. As the nutrients available to the bacterial cells will differ between growth conditions (e.g. niches in the human body, or growth media), gene essentiality will also differ between conditions. To clarify which genes are essential under which conditions, a genome-wide single guide RNA (sgRNA) library in which every known operon is targeted by an sgRNA, was developed for the D39V genome. For each of the 1498 unique sgRNAs in this library, the binding site position is now available in a separate genome-wide data track (Figure 3A) (12,13).

Using CRISPRi-Seq, this sgRNA library has been used to determine gene essentiality in a murine pneumococcal pneumonia (MPP) model, a murine pneumococcal pneumonia model with an influenza A virus superinfection (MPP + IAV), and under eight *in vitro* (infection-mimicking) growth conditions ((12), de Bakker and Veening, data to be published). For each transcribed feature, the essentiality under these conditions is given as either ‘essential’ or ‘non-essential’, per sgRNA targeting that feature (Figure 3B).

In addition, gene essentiality under *in vitro* infection-mimicking growth conditions, coupled transcriptomic and proteomic experiments have determined the transcriptional and translational activity for each coding feature under six distinct *in vitro* (infection-mimicking) growth conditions (de Bakker and Veening, data to be published). In PneumoBrowse 2, these data are added as transcripts per million (TPM) or proteins per million (PPM) to reflect their relative abundance (Figure 3B).

As previously noted, the use of ribosome profiling has led to the annotation of 122 new coding features in D39V. To clarify the annotation origin of these locus tags, we have included the ribosome profiling data that demonstrates the sites of active translation in three new genome-wide data tracks (14). These data contain tracks for control experiments and experiments in which retapamulin and lefamulin were used. Although normal conditions may already elucidate the position of the ribosome upon initiation of translation, the addition of retapamulin and lefamulin, two pleuromutilin-class antibiotics that stall translation and thus halt the ribosome after it has bound to an mRNA molecule, further aids the identification of previously unidentified start codons. The data tracks display the genome-wide normalized abundances of the RNA sequences to which ribosomes were bound. These values are displayed as mapping to the positive (red peaks; upwards) or negative (blue peaks; downwards) strands of the D39V genome (Figure 1A), according to the identified sequences.

### Improving gene function annotation by cross-referencing with PneumoWiki, UniProt and AlphaFold

The addition of genome-wide data tracks provides useful additional information about the expression and regulation of annotated features. However, these data cannot provide functional information of those features. To provide the best functional annotation possible, we have enriched the information for each coding feature with data from recent publications and several external databases. For example, the information for each gene now includes data on its expression and regulation (when available; Figure 3B) and the corresponding locus tags in other *S. pneumoniae* strains (D39 (SPD_XXXX), R6 (sprXXXX), and TIGR4 (SP_XXXX)) and the NCBI locus tag (RS_SPVXXXXX). In addition, we also provide cross-referencing to other databases: including KEGG and QuickGo (both extracted from eggNOG 5.0), PneumoWiki (https://pneumowiki.med.uni-greifswald.de), PneumoExpress (both expression, and co-expression data; (6)), Foldseek (59), the NCBI, UniProt, (52), AlphaFold (60,61) and STRING (62) (Figure 3C). For translated features, we provide a direct link to PaperBLAST using amino acid sequences (63).

### Expanding PneumoBrowse 2 with 18 additional genomes from phylogenetically diverse strains

**Figure 4.**
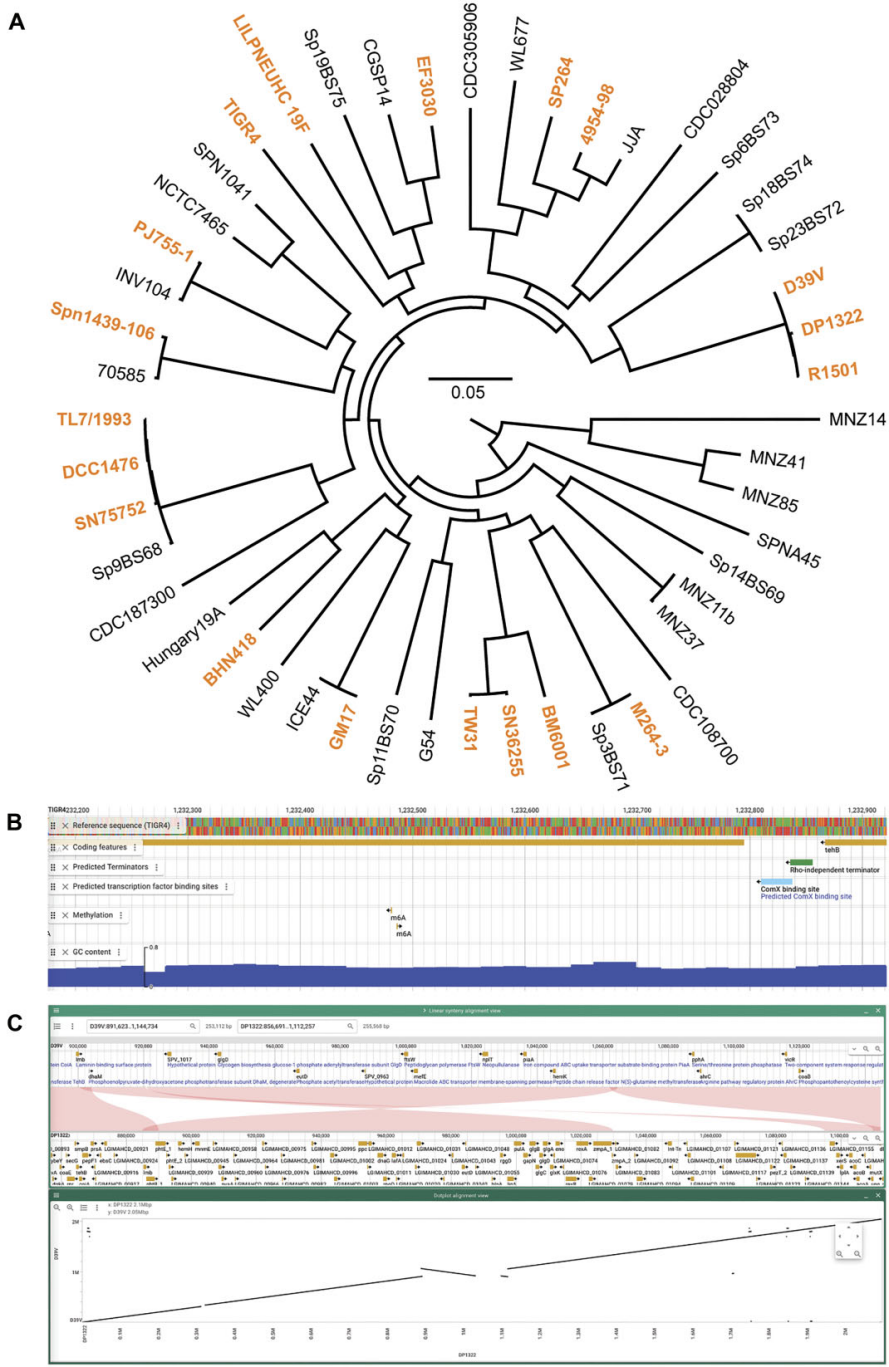
Addition of 18 phylogenetically diverse pneumococcal genomes besides D39V to PneumoBrowse 2. (**A**) A mid-point-rooted maximum likelihood phylogenetic tree based on the core genome alignment of the genome sequences presented here, and genomes representing the phylogenetic diversity within the *S. pneumoniae* species, based on a recombination-corrected core genome alignment (42). Genomes included in PneumoBrowse 2 are highlighted in orange boldface text. (**B**) For the 18 genomes besides D39V, tracks describing the following are available: the reference sequence; genomic annotation; predicted Rho-independent terminator sequences; predicted binding sites for ComE, ComX and ParB; and methylated genomic bases (not available for SN7572 and R1501). A track for the calculation (using customizable settings) of GC content is provided through the NucContent plugin. (**C**) Genome wide alignments between all genomes included in PneumoBrowse 2 are available through the data track from each individual genome panel, or through the linear synteny and dotplot alignment views panels. The linear synteny view and dotplot view of the alignment between D39V and DP1322 show the large inversion previously identified in D39V (5).

We have taken advantage of the multi-genome capability of JBrowse 2 by adding the genomic sequences of 18 other *S. pneumoniae* strains to PneumoBrowse 2 (Table 1). We performed long-read sequencing for the genomes of several oft-used strains, e.g. EF3030 (serotype 19F), TIGR4 (serotype 4) and BHN418 (serotype 6B), and for reference strains of several clones of the Pneumococcal Molecular Epidemiology Network (PMEN) (https://www.pneumogen.net/), including penicillin non-susceptible reference strains SP264 (PMEN18, serotype 14) and TW31 (PMEN14, serotype 19F). These genomes represent a phylogenetically diverse selection of *S. pneumoniae* strains (Figure 4A). For each of these 18 additional genomes, we provide the long-read assembled genomes in PneumoBrowse 2, as well as their genomic annotations, predicted transcriptional regulators binding sites, Rho-independent terminator sequences, and genomic base-modifications (Figure 4B). Within the annotation, enzyme classifiers (e.g. Prodigal classifiers and Enzyme Commission numbers) and links to AlphaFold, Foldseek and Uniprot are provided. To facilitate cross-genome comparison, we have added alignment data to PneumoBrowse 2. The alignments may be inspected from the individual genome panels, or through the linear synteny and dotplot alignment views panels (Figure 4C).

Although the strains presented in PneumoBrowse 2 are of a diverse phylogenetic standing within the *S. pneumoniae* species, some strains are observed to cluster within the same branch (Figure 4A). For example, the close clustering of D39V, DP1322 and R1501 is noteworthy, but is explained by their pedigree, as they originate from the D39 strain (4), which is a common model-strain for pneumococcal pathogenesis. The loss of the capsule locus by D39 led to the definition of the R6 branch, which later led to the creation of strain DP1322 (encoding Tn*5253*) and strain R1501 (harboring a *comC* deletion) (64). Prolonged cultivation and spread of D39 led to the definition of plasmid-free D39V (5). Likewise, the genomes of TL7/1993, DCC1476, and SN75752 (serotypes 9V, 15A and 11A, respectively) on one hand, and TW31 and SN36255 (serotypes 19F and 19A), on the other hand, are also observed to cluster together in two distinct branches. As strains SN36255 and SN75752 were isolated from infections of hospitalized patients, their likeness to reference strains for clinically relevant PMEN clones is not surprising. The other genomes are observed to be phylogenetically distinct.

Amongst the additional genomes is the genome of TIGR4, which was the first pneumococcal strain to have its genome fully sequenced by The Institute for Genomic Research (TIGR, now J. Craig Venter Institute; accession number NC_003028.3) (17). Several differences were identified through alignment of the genome determined by TIGR, and the one presented here. The differences in the repeat-rich genes *pavB* and *psrP* were deemed most significant. Within the TIGR genome, *pavB* is described to consist of four streptococcal surface repeats (SSUREs) (17). This contrasts to the five SSURE repeats we identify here. As for the number of repeats in *psrP*, a difference was observed in the second of the two serine-rich repeats (SRRs). The TIGR genome has been determined to have 539 (imperfect) repeats of the amino acid sequence SAS[A/E/V]SAS[T/I] in the SSR2 domain of PsrP, whereas we observe 225 (65). Besides the differences in these repeat-rich genes, no other changes in genetic content or structural differences were observed highlighting the high quality of the original TIGR4 genome sequence performed by shotgun Sanger sequencing.

## Discussion

In this work, we present an up-to-date iteration of the heavily used *S. pneumoniae* genome browser: PneumoBrowse 2. The continued development of novel methods to interrogate the *S. pneumoniae* genome is increasingly revealing the complex biology behind its regulation and organization. We have further refined the previous, already detailed, annotation of D39V by updating existing, adding new and removing redundant features as well as by adding DNA methylation sites, sgRNA bindings sites, quantitative coupled transcriptome and proteome data, gene essentiality, chromosomal protein binding and chromosome conformation data. Through the continued improvement of the annotation of its genomic content, the increasingly detailed annotation of D39V will continue to set the standard for the annotation of other genomes from both *S. pneumoniae* and other bacterial species.

Previous efforts to understand the regulation and contents of the *S. pneumoniae* D39V genome relied on RNA and DNA sequencing. However, these methods have not been able to fully determine the D39V proteome due to inherent challenges linked to transcript abundance, and computational algorithms. New work using ribosome profiling has led to the annotation of 122 previously undescribed sORFs, typically less than 50 amino acids in size (14). The small size does not mean that these sORFs cannot have a substantial impact on pneumococcal biology (66). For example, the competence-stimulating peptide of *S. pneumoniae* plays a crucial role in the regulation of competence and transformation, yet measures only 17 amino acids in length (67). As only eight sORFs have had their function (partly) presently elucidated, a vast potential for the discovery of novel biological mechanisms remains.

Besides updated D39V annotation, we extend the use of PneumoBrowse to a wider audience by providing the genomic sequence and annotations of 18 additional, phylogenetically diverse, *S. pneumoniae* strains, including that of TIGR4. The observed differences in two well-known repetitive regions (in *pavB* and *psrP*) between the TIGR4 genome determined by Tettelin *et al.* (17) and the one presented here, may either represent natural variation in closely related strains that has arisen through recombination events or mishaps during genome duplication or be the consequence of different genome sequencing techniques and assembly algorithms resulting in differences in these difficult-to-solve regions (68–70). Such variations have been noted before, as a different number of repeats in *pavB* of TIGR4 than the genome published by Tettelin *et al.*, have been reported before (71). These (and other) variations influence the exact genomic coordinates between these genomes.

Since the introduction of PneumoBrowse 1, other web-based resources that offer accession to genome annotations have also been developed. These databases (e.g. PneumoWiki (https://pneumowiki.med.uni-greifswald.de), the KEGG database, the NCBI Nucleotide genome browser, and the BioCyc Genome Explorer (40,72–74)) have been designed to follow different philosophies than PneumoBrowse. PneumoBrowse offers the intuitive visualization of the annotation and other data types of a variety pneumococcal genomes, to distinguish itself as a genome browser. We anticipate that the free availability of the annotations presented here will increase understanding of the *S. pneumoniae* strains.

## Supplementary Material

gkae923_Supplemental_Files

## Data Availability

All long-read sequencing data, and base modification files for PacBio sequenced genomes (except for D39V), are available in the NCBI Sequence Read Archive (75) under BioProject accession code PRJNA1103744. Assembled genomic sequences are available from NCBI through the respective accession codes (Table 1). Genome sequence and methylation data from D39V are available under BioProject accession code PRJNA295913 (5). The SN75752 genomic sequence is available under accession code CP089949.1 (76). PneumoBrowse 2 is freely available on https://veeninglab.com/pneumobrowse.
